# Progressive Muscle Weakness: Identifying Necrotizing Autoimmune Myopathy as a Rare Culprit

**DOI:** 10.7759/cureus.79164

**Published:** 2025-02-17

**Authors:** Bojan Miletic, Simon Schneiter, Stavroula Lygkoni

**Affiliations:** 1 Department of Geriatrics and Rehabilitation, Lucerne Cantonal Hospital Wolhusen, Wolhusen, CHE; 2 Clinical Medical Science, Faculty of Health Studies, University of Rijeka, Rijeka, HRV; 3 Department of Internal Medicine, Lucerne Cantonal Hospital Wolhusen, Wolhusen, CHE; 4 Department of Cardiology, University Hospital of Zürich, Zürich, CHE

**Keywords:** complication, pathophysiology, statin-induced necrotizing autoimmune myopathy, statin use, treatment choices

## Abstract

Statin-induced necrotizing autoimmune myopathy (SINAM) is a rare but critical complication of statin therapy that leads to progressive muscle weakness. The complicated mechanisms underlying it make both diagnosis and treatment difficult. It is essential to recognize the condition early, especially in people who have had muscle problems treated with statins in the past. A 73-year-old woman arrived at the emergency department due to increasingly severe symmetrical muscle weakness accompanied by markedly elevated liver enzymes, creatine kinase (CK), and cardiac troponins. Although an acute cardiac event was ruled out, further tests indicated progressive myositis, necessitating hospitalization. A muscle biopsy subsequently confirmed myopathy with complement deposition, and anti-3-hydroxy-3-methylglutaryl-coenzyme A reductase (anti-HMGCR) antibodies were detected. With treatment that included corticosteroids, intravenous immunoglobulin, and rehabilitation, the patient showed remarkable improvement. This case undeniably highlights the critical importance of early detection of SINAM and intervention and emphasizes the absolute need for further research into causes and treatment strategies.

## Introduction

Statin-induced necrotizing autoimmune myopathy (SINAM) is a significant and serious complication of statin use that should not be overlooked. Although most side effects of statin therapy are mild, SINAM is a rare but serious condition that occurs in approximately 2 to 3 per 100,000 users [1]. However, in their 2020 case report, Gawey and colleagues estimated the incidence to be only 1 in 100,000 cases, which is probably due to the still relatively low number of recognized and published SINAM cases [2]. Several authors emphasize age over 50 years as a risk factor as well as African-American ethnicity and stress the possible immunogenetic association with certain HLA alleles [3-5]. They also point to other risk factors such as uncontrolled diabetes and liver and kidney disease. As a systematic review by Somagutta et al. shows, which analyzed a total of 80 cases of SINAM published between 2010 and 2022, the disease predominantly affects men. Their results showed that 61.3% of patients were male, mainly older adults with an average age of 65.9 years [1]. These data are in line with other studies, such as that of Nazir et al. (2017), who examined a total of 100 cases [6]. One possible explanation is the higher prevalence of cardiovascular disease in men and consequently the more frequent use of statin therapy, which is confirmed by numerous studies. However, all these facts show that there are still many unknowns about SINAM. For example, the exact cause of SINAM is still unclear, and the symptoms can occur at any time after starting statin therapy and can persist for a long time after stopping statins. This unpredictability poses a major challenge for both diagnosis and treatment. Healthcare providers must be extremely vigilant when evaluating patients with progressive muscle weakness, as early detection and intervention are critical. This case report addresses the potential causes, diagnostic challenges, and treatment options for SINAM and emphasizes the importance of prompt recognition and clinical intervention.

## Case presentation

A 73-year-old woman presented to the emergency department because her muscle weakness and leg pain had gradually worsened in recent weeks. She was currently taking candesartan, amlodipine, and bisoprolol, as she was under treatment for arterial hypertension for several years. She was also treated with atorvastatin (40 mg), but after five months, the drug was discontinued due to elevated liver enzyme levels, although she had no muscle-related symptoms. Later, rosuvastatin (20 mg) was included in the therapy, but this drug was also discontinued three months later for the same reason. However, due to the patient's mild cognitive impairment, the anamnestic data on statin intake and progression of symptoms could not be considered completely reliable. On physical examination, she had proximal leg muscle weakness, rated 4 on the Medical Research Council (MRC) muscle strength scale, which slightly limited her ability to perform activities of daily living. Her peripheral sensation was intact. Routine laboratory tests revealed elevated levels of aspartate aminotransferase (AST), alanine aminotransferase (ALT), lactate dehydrogenase (LDH), and the isoenzyme of creatine kinase (CK-MB), and a significant increase in creatine kinase (CK) as well as an elevated high-sensitivity cardiac troponin (hs-cTnT) (Table 1).

**Table 1:** Laboratory findings of the patient AST - Aspartate aminotransferase, ALT - Alanine aminotransferase, LDH - Lactate dehydrogenase, CK - Creatine kinase, CK-MB – Creatine kinase-MB isoenzyme, hs-cTnT - High-sensitivity cardiac troponin

Parameter	Day 1	Day 20	Reference Range
AST	115 U/L	32 U/L	< 33 U/L
ALT	171 U/L	35 U/L	< 33 U/L
LDH	1702 U/L	170 U/L	140-280 U/L
CK	4830 U/L	279 U/L	30-140 U/L
CK-MB	>300 µg/L	2 µg/L	<4.88 µg/L
hs-cTnT	405 ng/L	4 ng/L	< 14 ng/L

**Figure 1:**
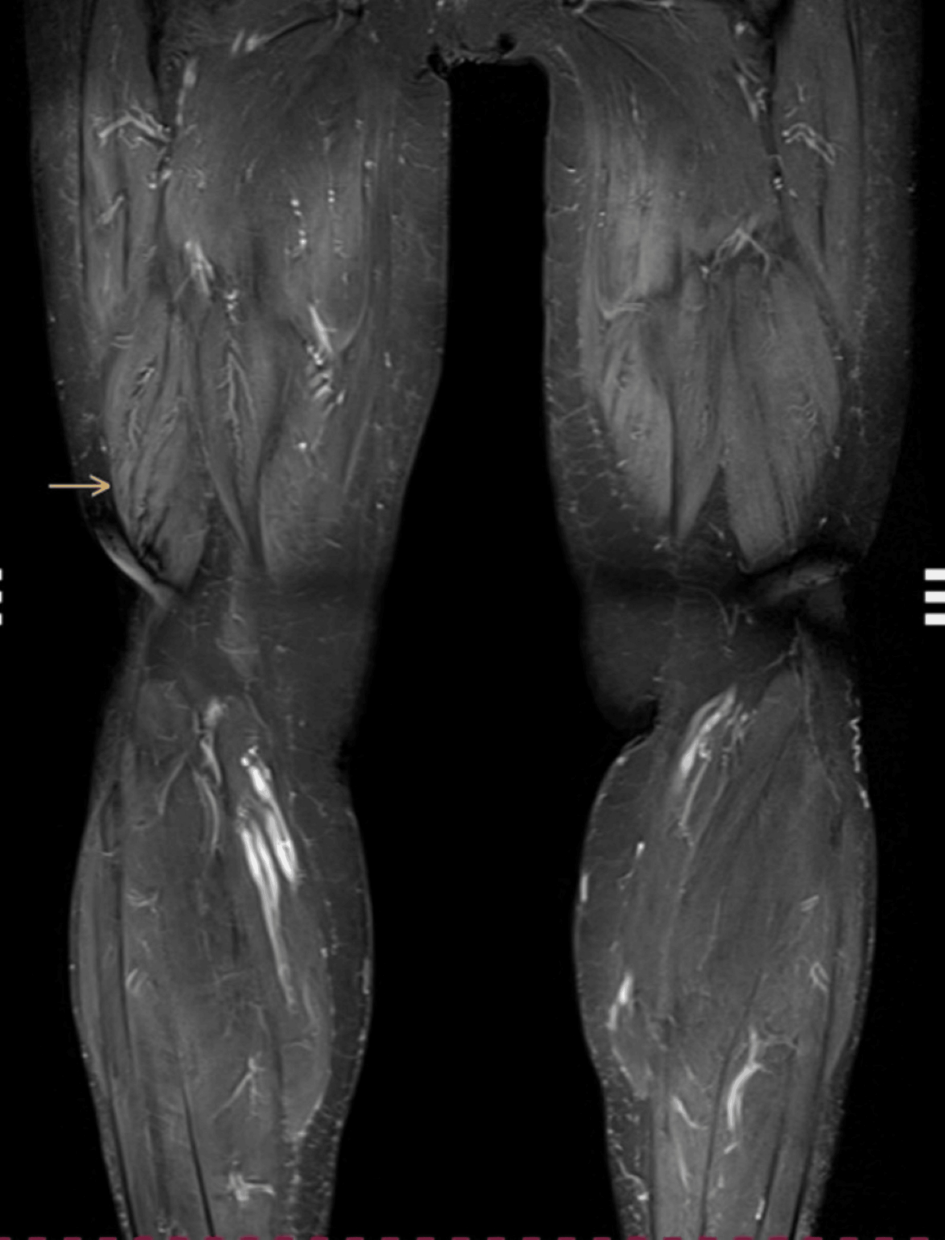
MRI of the legs The yellow arrow indicates an altered T2 signal in multiple muscles, consistent with myositic changes.

However, other parameters, such as blood count, C-reactive protein, renal function markers, electrolytes, albumin, amylase, and lipase, were all within the normal range. Aldolase was not tested for technical reasons. Due to the abnormal troponin levels, considering the possibility of an acute cardiovascular event or inflammatory myocardial disease, further cardiac investigations were performed, including ECG, echocardiography, and cardiac MRI, but no abnormalities were detected. Given the differential diagnostic possibility of a symptom presentation in the context of a paraneoplastic syndrome, a CT scan of the head, neck, chest, and abdomen including the analysis of tumor markers was performed, which revealed no pathological findings. Given that statin therapy had been discontinued three months earlier and the current progression of muscle weakness in the legs, an MRI of the lower extremities was performed, which showed changes in T2 signal intensity in several muscles, with the most pronounced abnormalities seen in the right rectus femoris, indicating myositis (Figure 1).

**Figure 2:**
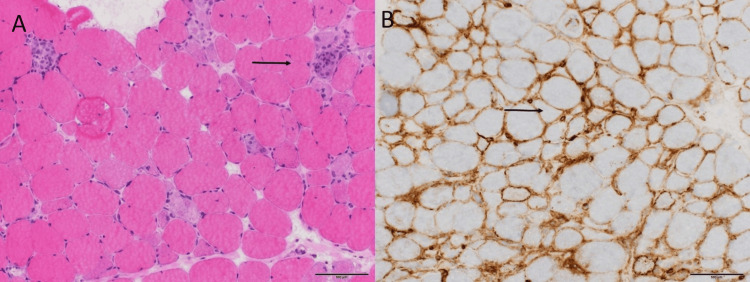
Hematoxylin and eosin staining image showing myophagy - macrophages phagocytizing necrotic muscle fibers (A) and immunohistochemical staining with antibodies against the membrane attack complex with complement deposition (B) at low magnification of 2x

This was followed by hospitalization for definitively planned investigations and a thorough clarification of the cause of the myositic changes observed on MRI, including various forms of idiopathic inflammatory myopathies. Because of the patient's age, her mild cognitive impairment, and her lonely living situation, the social component was also clearly taken into account. Autoimmune serologic testing for various markers according to the hospital's standard protocol, including ANA, Jo-1, U1-snRNP, Sm, anti-Mi2, anti-TIF1γ, anti-MDA5, anti-NXP2, anti-SAE1, anti-Ku, anti-PM-Scl, anti-SRP, anti-PL7, anti-PL12, anti-EJ, anti-OJ, and anti-Ro52, all yielded negative results. HLA-DRB1 typing was not performed for technical reasons, although this data could help understand the patient's overall genetic risk for autoimmune myopathies. A biopsy of the right rectus femoris muscle revealed the following: the HE-stained image showed numerous myophagia (macrophages phagocytizing necrotic muscle fibers) as well as one acutely necrotizing muscle fiber without macrophage infiltration or myophagia at this stage. In addition, there was an unusually strong complement deposition along the sarcolemma in almost all muscle fibers, as shown by immunohistochemical staining with antibodies against the membrane attack complex C5b-9 (Figure 2).

Further analysis confirmed a positive level of antibodies against anti-3-hydroxy-3-methylglutaryl-coenzyme A reductase (anti-HMGCR). Considering the symptoms, medical history, and diagnostic findings, the patient was diagnosed with SINAM. One week after the start of therapy, which included corticosteroids (prednisone 30 mg daily, with gradual dose reduction), intravenous immunoglobulin therapy (0.5 g/kg/day) for five days, and physiotherapy, the patient showed rapid improvement. Three weeks later, when she was discharged, her laboratory values had improved significantly. Repeated functional tests, such as the FIM (Functional Independence Measure) score, the Tinetti Balance and Gait Assessment, the Six-Minute Walk Test, and the Timed Up and Go Test, also showed significant improvement. The patient was discharged from the hospital with organized social and medical support at home. Unfortunately, the patient did not attend any follow-up appointments, making it impossible to monitor her clinical condition and treatment.

## Discussion

Statins are widely used to prevent cardiovascular disease and their role in primary and secondary prevention has increased considerably. According to Mortensen et al., the use of statins nearly doubled between the 2016 and 2019 guidelines [7]. While this expansion improves the prevention of cardiovascular disease, it also raises concerns about potential side effects. One of the possible side effects of taking statins is progressive muscle weakness, which is often associated with elevated CK values. In such cases, several diseases must be considered, including polymyositis, dermatomyositis, and metabolic myopathies [8]. However, in our case, the patient’s autoimmune tests for markers such as ANA and Jo-1 were negative, making polymyositis and dermatomyositis less likely. Metabolic myopathies are caused by defects in the body’s metabolism, such as problems with fat metabolism, and often lead to muscle weakness, especially after exercise or fasting. These conditions usually have a more specific pattern of muscle weakness or associated metabolic problems, such as abnormal blood glucose or lipid levels, which were not present in this patient. Myositis can also be caused by infectious agents, but in this case, the patient's medical history was negative for any signs of infection [8]. Given the patient’s history of statin use and the unique findings, SINAM seemed the most likely diagnosis. This rare but serious complication of statin therapy fit the clinical picture and was ultimately considered the best explanation for the patient’s symptoms. Although it is often difficult at first glance to distinguish the symptoms of SINAM from other forms of inflammatory myopathies, such as dermatomyositis or polymyositis, SINAM is characterized by its rare extramuscular manifestations, the presence of positive anti-HMGCR antibodies and specific pathological changes, including complement deposition on capillaries and/or the sarcolemma. Muscle-related side effects affect about 10% to 25% of patients taking statins and include all muscle-related symptoms, from mild myalgias to the most severe forms of disorders [9]. The exact cause of SINAM is still not fully understood, but researchers suspect a link to cholesterol production disorders, mitochondrial dysfunction, or reduced cholesterol levels in muscle cells [10]. Some factors may increase the risk, including older age, lower body mass index, females, and alcohol consumption. The role of vitamin D in SINAM remains a topic of ongoing debate, but the meta-analysis by Hou and colleagues showed that low levels of 25-hydroxyvitamin D are linked to statin-related myopathy and that vitamin D supplementation can improve muscle intolerance. This topic will certainly be the subject of further research due to the potential benefits of vitamin D in the prevention and treatment of statin-induced myopathies [11]. Research on statin types and doses suggests that lipophilic statins (atorvastatin, fluvastatin, lovastatin, simvastatin) may have higher myotoxicity unlike hydrophilic statins (pravastatin, rosuvastatin). Lipophilic statins can easily penetrate the muscle cell membrane due to their lipophilicity. This penetration contributes to a higher probability of muscle-related side effects. Hydrophilic statins, on the other hand, are more water soluble and therefore have a harder time penetrating the cell membrane and entering the cell [9]. Essers et al. linked SINAM predominantly with lipophilic statins [12]. Statin interactions with drugs metabolized by the same enzymes, particularly CYP3A4, may also contribute to adverse effects [13]. Mild symptoms might have been overlooked initially, as Mohassel et al. reported that up to 20% of patients improve after stopping statins [14]. In some cases, however, symptoms persist, indicating a strong immune response and the need for long-term monitoring of these patients [1]. Symptoms usually appear within 10 years of statin initiation, though cases with onset up to 20 years later have been documented. Clinical signs include progressive muscle weakness, arthralgia, dysphagia, skin rash, and Raynaud’s phenomenon, often with significantly elevated CK levels. Anti-HMGCR antibodies are important diagnostic markers for SINAM, but they can also occur in people who are not taking statins. Studies have shown that these antibodies have been found in both children and adults who have not received statin therapy or have cancer, as well as in patients with other autoimmune diseases such as rheumatoid arthritis or systemic lupus erythematosus and idiopathic inflammatory myopathies [15]. Various methods are used to identify anti-HMGCR antibodies, with the enzyme-linked immunosorbent assay (ELISA) being the standard diagnostic tool. It has a sensitivity of 94.4% and a specificity of 99.3% [16]. However, the possibility of false positive and false negative results makes it clear that, in addition to the test results, clinical interpretation must also be a key factor in the diagnosis. Anti-HMGCR antibodies are directed against the enzyme HMGCR, which is involved in the production of cholesterol in the liver. Statins inhibit this enzyme and thus reduce cholesterol production. However, they can also trigger an immune reaction that leads to the formation of anti-HMGCR antibodies. These antibodies activate the complement system and form membrane attack complexes (C5b-9) on the muscle cell membrane. This leads to muscle damage, elevated CK levels, and other laboratory abnormalities, with progressive muscle weakness often affecting the proximal muscles. Although the exact mechanism is not yet fully understood, genetic factors are likely to play an important role. Studies suggest a possible association with certain HLA alleles, particularly HLA-DRB1, as shown in the study by Kishi et al. [17]. Diagnosing SINAM involves clinical assessment, including a history of statin use, muscular symptoms, muscle biopsy, laboratory tests, and imaging studies. Ultrasound is a convenient alternative to bedside evaluation of muscle inflammation because it can detect muscle edema and inflammation and, in the hands of an experienced ultrasonographer, can detect muscle atrophy and fibrosis in chronic disease. Given the association between myositis and malignancies, CT imaging is often used in paraneoplastic investigations, which is why a comprehensive CT scan was performed in this case. However, the standard for imaging is still MRI, as it is highly sensitive to detect structural changes, edema, and inflammation in the affected muscles and helps to differentiate between various types of myositis [18]. A characteristic pathohistological finding is muscle fiber necrosis with inflammatory components and immune complex deposition in unaffected fibers, aligning with our case [1]. T2 hyperintensity on MRI reflects active muscle inflammation/necrosis while complement deposition is an important diagnostic marker indicating immune-mediated muscle damage in SINAM. The complexity of SINAM makes treatment challenging.

HLA-DRB1 typing was not performed in this case. This could have contributed to a better understanding of the patient’s predisposition to SINAM. In general, the timely identification of individuals at higher risk for SINAM is crucial for assessing the risk-benefit ratio of statin therapy. In such situations, a more cautious approach to the use of statins or the choice of alternative cholesterol-lowering drugs (such as PCSK9 inhibitors), if necessary, may help reduce the risk of an autoimmune reaction. In any case, these patients require closer monitoring with more frequent clinical and laboratory tests to detect early signs of muscle weakness or elevated enzyme levels. Current treatment strategies include corticosteroids and intravenous immunoglobulins, either alone or in combination. However, specific treatment plans and duration of therapy have not yet been defined. Therefore, according to some authors, methotrexate, azathioprine, or mycophenolate mofetil are possible alternatives that could help reduce the side effects associated with long-term steroid use. These could be used in patients who cannot tolerate steroids or in cases where patients do not respond well to the discontinuation of statins or corticosteroids [15]. This case report highlights the delayed onset of SINAM after discontinuation of statins, which contrasts with the immediate onset in other reports. The diagnostic process was particularly complex as various possible causes (such as cardiac problems or paraneoplastic syndrome) had to be considered. The report also emphasizes the importance of considering social and mental factors in patient care, especially in older adults. It should be recognized that the lack of genetic testing is a missing piece of the diagnostic puzzle, as it could have provided additional information and helped determine treatment options. In addition, the inability to follow up with the patient and the onset of symptoms prevented an evaluation of the effectiveness of the treatment. Nevertheless, this article aims to raise awareness of the rare side effects of statin therapy, SINAM, and the challenges in diagnosis. It emphasizes the need for further research into the pathophysiological mechanisms of the disease and its genetic basis, which would help define treatment strategies more precisely. Despite these challenges, timely diagnosis, which must include adequate patient education for early recognition of symptoms, remains a crucial element for successful treatment.

## Conclusions

As muscle weakness is a common early symptom, SINAM should be considered in the differential diagnosis of patients who have taken statins in the past. Further research is needed to develop evidence-based treatment guidelines and to better understand the pathogenesis of SINAM. This case underscores the importance of clinicians becoming aware of this rare but serious side effect of statin therapy. This is particularly important given the increasing use of statins for primary and secondary prevention, which may lead to an increase in associated side effects. The case also highlights the need for regular clinical and laboratory monitoring of patients receiving statin therapy, as well as the identification of patients at higher risk to detect potential complications early and initiate appropriate treatment.
